# Determinants of Patients’ Intention to Use the Online Inquiry Services Provided by Internet Hospitals: Empirical Evidence From China

**DOI:** 10.2196/22716

**Published:** 2020-10-29

**Authors:** Dehe Li, Yinhuan Hu, Holger Pfaff, Liuming Wang, Lu Deng, Chuntao Lu, Shixiao Xia, Siyu Cheng, Ximin Zhu, Xiaoyue Wu

**Affiliations:** 1 School of Medicine and Health Management Tongji Medical College Huazhong University of Science and Technology Wuhan, Hubei China; 2 Center for Health Services Research Cologne University of Cologne Cologne Germany; 3 Tongji Hospital Tongji Medical College Huazhong University of Science and Technology Wuhan, Hubei China; 4 Jingmen No. 2 People’s Hospital Jingmen, Hubei China

**Keywords:** internet hospital, health care–seeking intention, online inquiry, theory of planned behavior, chronic disease, structural equation modeling, China, COVID-19, intention, online service, eHealth, behavior, modeling

## Abstract

**Background:**

Internet hospitals show great potential for adequately fulfilling people’s demands for high-quality outpatient services, and with the normalization of the epidemic prevention and control of COVID-19, internet hospitals play an increasingly important role in delivering health services to the public. However, the factors that influence patients’ intention to use the online inquiry services provided by internet hospitals remain unclear. Understanding the patients’ behavioral intention is necessary to support the development of internet hospitals in China and promote patients’ intention to use online inquiry services provided by internet hospitals during the prevention and control of the COVID-19 epidemic.

**Objective:**

The purpose of this study is to identify the determinants of patients’ intention to use the online inquiry services provided by internet hospitals based on the theory of planned behavior (TPB).

**Methods:**

The hypotheses of our research model were developed based on the TPB. A questionnaire was developed through patient interviews, verified using a presurvey, and used for data collection for this study. The cluster sampling technique was used to include respondents with chronic diseases. Structural equation modeling was used to test the research hypotheses.

**Results:**

A total of 638 valid responses were received from patients with chronic diseases. The goodness-of-fit indexes corroborated that the research model was a good fit for the collected data. The model explained 45.9% of the variance in attitude toward the behavior and 60.5% of the variance in behavioral intention. Perceived behavioral control and perceived severity of disease had the strongest total effects on behavioral intention (β=.624, *P*=.004 and β=.544, *P*=.003, respectively). Moreover, perceived convenience, perceived information risk, emotional preference, and health consciousness had indirect effects on behavioral intention, and these effects were mediated by attitude toward the behavior. Among the four constructs, perceived convenience had the highest indirect effect on behavioral intention (β=.207; *P*=.001).

**Conclusions:**

Perceived behavioral control and perceived severity of disease are the most important determinants of patients’ intention to use the online inquiry services provided by internet hospitals. Therefore, internet hospitals should further optimize the design of online service delivery and ensure a reasonable assembly of high-quality experts, which will benefit the promotion of patients’ adoption intention toward online inquiry services for health purposes. Perceived convenience, emotional preference, and perceived risks also have effects on behavioral intention. Therefore, the relevant quality control standards and regulations for internet hospitals should be further developed and improved, and the measures to protect personal information should be strengthened to ensure the patient safety. Our study supports the use of the TPB in explaining patients’ intention to use online inquiry services provided by internet hospitals.

## Introduction

### Background

With the development of human society, an aging population with chronic and age-related diseases, and its increasing health care requirements have become a challenge for the governments of all countries worldwide, especially China [1,2]. In China, many people complain about the “difficulty of seeing a doctor” and tend to go to high-level hospitals even for mild symptoms [3]. It is difficult for patients in rural and remote areas to access high-quality health services owing to an uneven distribution of medical resources [4,5]; moreover, the time spent by physicians on communicating with each patient is limited, usually less than 3 minutes in outpatient departments in large hospitals [6]. A latest national health services survey showed the rate of chronic diseases in Chinese residents was up to 245.2 person per thousand population, and the majority of the common chronic diseases were hypertension, diabetes, and heart disease [7]. Patients with chronic diseases such as diabetes receive little education or information about their diseases in such a short time [8]; thus, their health service requirements are rarely fulfilled. Under such circumstances, inability of traditional medical resources to meet the increasing demands of health services for the public contributes to the emergence of internet hospitals [9].

Internet hospitals, a novel approach to health service delivery via internet technology [3,9,10], were developed as a potential solution to optimize the allocation of health resources and to meet people’s demands for high-quality health services [3]. Compared with mobile health (mHealth), a technology integration of mobile computing, medical sensors, and portable devices for meeting the needs of consumers about health care or health information services [11], internet hospitals refer to one-stop online service platforms based on traditional hospitals, which integrate consultation, prescription, payment, and drug delivery; internet hospitals enable patients to communicate with skilled experts anytime and anywhere using a website or a smartphone app and gain access to health-related services such as online disease counseling, online guidance, electronic prescriptions, medical information, remote diagnosis, treatment and rehabilitation, chronic disease follow-up, and health education [3,9]. Several studies have shown that compared with traditional medical services, internet hospitals increased the time that the physicians spent with each patient, improved the accessibility to high-quality health care, alleviated the difficulty of “seeing a doctor” [3,12], promoted the rational distribution of health resources, and improved the balance between supply and demand of health resources in China [4,9]. In addition, the Chinese government has implemented an ambitious national strategy called “Health China” since 2016, and this strategy is expected to solve the problem of “difficulty and expense of seeing a doctor” and improve the ability of medical resources to meet the increasing demands of the public health services in China [13]. Thus, the development of internet hospitals is crucial for implementing this national strategy.

Internet hospitals have also shown great potential for the epidemic prevention and control of COVID-19 in China [10,14]. During the COVID-19 epidemic, internet hospitals, which provide free COVID-19 consultation services and offer essential medical support to the public, have effectively reduced the probability of nosocomial cross-infections and fulfilled patients’ demands for outpatient services, especially for patients with chronic diseases [10], and thus, have been further widely accepted by society. However, a latest research report in China in 2020 has highlighted that the users’ acceptance of online inquiry services provided by different types of internet hospitals was relatively poor, with significant differences [15]. Among enterprise-led internet hospitals such as WeDoctor, Hao Yisheng, and AliHealth, more than half (53.4%) provided services to less than 1000 patients per day, whereas approximately one-third (26.6%) provided services to more than 3000 patients per day. Moreover, the number of daily outpatient visits of medical institution-led internet hospitals such as the internet hospital of People’s Hospital of Zhejiang Province and the internet hospital of No.2 People’s Hospital of Guangdong Province was generally lower, with two-thirds (66.7%) of the internet hospitals providing services to less than 100 patients per day.

With the normalization of prevention and control of the COVID-19 epidemic, internet hospitals play an increasingly important role in delivering health services to the public. Moreover, issues such as whether patients accept internet hospitals and what factors influence patients’ decisions regarding the online inquiry services provided by internet hospitals should be considered for the long-term development of internet hospitals. Understanding the factors that affect patients’ intention to use online inquiry services will help internet hospitals to further improve their service design, promote their development, and thus promote patients’ health care–seeking intention to use online inquiry services during the prevention and control of the COVID-19 epidemic. However, the factors influencing patients’ health care–seeking intention to use online inquiry services provided by internet hospitals remain unclear.

Previous studies on patients’ health care–seeking behaviors of online inquiry focus on mHealth services, and theoretical models have been applied to identify the determinants of adoption intentions for mHealth services [8,13,16-18] or health technology [19-21]; however, few studies focus on patients’ health care–seeking intention to use the online inquiry service provided by internet hospitals. A theoretical model must be identified and tested to provide a context-related understanding of patients’ intention to use the online inquiry services provided by internet hospitals. Internet hospitals have unique service delivery characteristics, and patients exhibit different degrees of acceptance for online inquiry services for different types of internet hospitals [15]. Thus, it is necessary to analyze the factors influencing the intention to use the online inquiry services provided by internet hospitals. To date, relevant theoretical models have not been applied to patients’ intention to use the online inquiry services in the context of internet hospitals.

### Theory of Planned Behavior

The theory of planned behavior (TPB), an expansion of the theory of reasoned action, has been widely used as a classical theory in explaining and predicting various types of human behaviors [22,23]. The TPB mainly consists of five variables: attitude toward the behavior, subjective norm (SN), perceived behavioral control (PBC), behavioral intention, and actual behavior. According to TPB, whether individuals perform a certain behavior depends on their behavioral intention to perform the behavior. Behavioral intention is determined by the following factors: attitude toward the behavior, SN, and PBC, which is also called adoption intention [23]. Compared with the technology acceptance model, which has been widely applied to the acceptance and use of information technology [13,16], TPB considers both the subjective and social factors affecting patients’ behavioral intention as well as external control conditions that are not affected by personal willingness [22,23]. In health care settings, patients’ intention to perform a behavior for health purposes is often the result of an interaction between internal and external factors. The TPB has been frequently applied in the field of mHealth services [24-27], and these studies found that attitude toward the behavior, SN, and PBC are the major determinants of behavioral intention toward mHealth service adoption. However, to date, TPB has not been used in the study of the factors influencing patients’ behavioral intention to use online inquiry services in the context of internet hospitals. Considering the uniqueness of mHealth service, Zhang et al [27] proposed the integration of TPB and protection motivation theory in the context of mHealth services and found an indirect effect of perceived severity of disease (PSD) on behavioral intention. Thus, patients’ PSD should be considered. To date, China has not established an effective quality control system for internet hospitals [12,15]. Potential medical risks of the online inquiry services provided by internet hospitals threaten patients’ health safety. Therefore, medical risks should also be considered.

Thus, in this study, we combined the TPB and patients’ PSD and perceived medical risks (PMRs) to develop a theoretical model to identify the factors influencing patients’ behavioral intention to use the online inquiry services provided by internet hospitals.

### Research Hypotheses and Model

In this study, the TPB model was adopted as the theoretical framework, where attitude toward the behavior refers to the extent to which a patient has a favorable or unfavorable opinion of online inquiry behavior; SN is defined as a patient’s perceived social pressure when performing online inquiry; and PBC is defined as a patient’s perception of the degree of ease or difficulty when using the online inquiry services provided by internet hospitals, and it is generally equivalent to self-efficacy [23,27]. Several studies have shown that attitude toward the behavior, SN, and PBC are the positive determinants of patients’ adoption intention toward mHealth services [24,26,27]. Therefore, three hypotheses were formulated as follows:

- Hypothesis (H)1: Attitude toward the behavior has a positive effect on the behavioral intention of patients to use the online inquiry services provided by internet hospitals.
- H2: PBC has a positive effect on the behavioral intention of patients to use the online inquiry services provided by internet hospitals.
- H3: SN has a positive effect on the behavioral intention of patients to use the online inquiry services provided by internet hospitals.

A previous study on the use of mobile technology demonstrated that perceived convenience (PC) positively affected attitude toward the behavior [28]. Internet hospitals not only save the time and effort that patients spend on seeking health services but also conveniently provide patients with health services via the internet anywhere and anytime. In this study, we define PC as patients’ perceptions of saving time and effort when using the online inquiry services provided by internet hospitals. If patients find it convenient to access health service while having to spend less time and effort, they will have a positive attitude toward participating in telemedicine [29]. Thus, we posed the following hypothesis:

- H4: PC has a positive effect on the attitude of patients toward using online inquiry in internet hospitals.

A study concerning mobile commerce demonstrated the effect of perceived outcome (PO) on behavioral intention, and this effect was mediated by behavioral attitude [30]. In health care settings, the value of PO affects patients’ choices to a certain extent, and patients in China tend to go to high-level hospitals rather than primary health care institutions even for mild symptoms [3]. If patients find that the service outcomes obtained from internet hospitals are consistent with or even better than those from traditional large hospitals, they may have a positive attitude toward online inquiry. In this study, PO refers to patients’ perceptions of the degree to which online inquiry achieves the expected health outcome improvement. Thus, we posed the following hypothesis:

- H5: PO has a positive effect on the attitude of patients toward online inquiry in internet hospitals.

Although mHealth services may promote the accessibility of high-quality health services, alleviate the difficulty in “seeing a doctor,” and thus improve patients’ quality of life, they also lead to issues regarding quality control, information security, and privacy protection [31,32]. Likewise, internet hospitals were potentially puzzled by these issues. The potential risks of using the online inquiry services provided by internet hospitals include medical risk and information risk. Patients may want to gain access to health services from internet hospitals but may not want to get involved in medical disputes for undesired outcomes or disclose their personal information resulting in online fraud. To date, China has not established an effective quality control system for internet hospitals [12,15], and their potential medical risks threaten patient safety, thereby possibly affecting patients’ intention to use the online inquiry services of internet hospitals. In this study, we define perceived information risk (PIR) as patients’ perception of lack of control over their personal information (ie, privacy information on health care–seeking data) after they have adopted the online inquiry services provided by internet hospitals. Information privacy concern is a core predictor of PIR [33]. A study by Deng et al [13] found that privacy risk was negatively correlated with adoption intention toward mHealth services, and a study by Zhang et al [8] on the intention to use diabetes management apps found that perceived privacy risk negatively influenced behavioral intention. Therefore, we formulated the following hypotheses:

- H6a: PMR has a negative effect on the behavioral intention of patients to use online inquiry in internet hospitals.
- H6b: PIR has a negative effect on the attitude of patients toward online inquiry in internet hospitals.

Emotional preference (EP) could be defined as emotions that individuals are motivated to experience; these emotions strongly influence various human behaviors [34]. In health care settings, a patient’s EP needs to be respected and be inaccessible protection from society. Several studies regarding patients with mental health problems suggest that the requirements of patients associated with their EPs, owing to issues such as stigma and social discrimination, affected their intention toward seeking help in time [35,36]. In internet hospitals, patients equipped with mobile devices can communicate with physicians without the constraints of time and space, which help to meet the requirements associated with their EPs, for example, privacy protection and avoiding embarrassment and stress to a certain extent; thus, those with EP may have a positive attitude toward online inquiry services of internet hospitals. Therefore, we established the following hypothesis:

- H7: EP has a positive effect on the attitude of patients toward online inquiry in internet hospitals.

With the rapid development of internet hospitals, the issue of their medical liability comes to the fore, threatening patient safety [3,37,38]. In addition to patients, physicians, and hospitals, the stakeholders of internet hospitals also involve a third-party medical platform, which could cause a more complex medical dispute. However, to date, the complaint system established by the Chinese government for internet hospitals regarding medical accidents and medical disputes is imperfect, and the related regulations from traditional hospitals are often used for the management of medical behaviors in internet hospitals [12,15]. If patients find it challenging to defend their rights and interests in internet hospitals, they may have a negative attitude toward online inquiry. Therefore, we posed the following hypothesis:

- H8: Perceived medical liability (PML) has a negative effect on the attitude of patients toward online inquiry in internet hospitals.

Health consciousness is defined as the degree to which health concerns are integrated into an individual’s daily life, thus creating a motivation for health improvement [39]. A study by Guo et al [40] found such a moderation effect of health consciousness on behavioral attitude toward continuous use of mHealth services [40], and an investigation by Ahadzadeh et al [20] found that the partial effect of health consciousness on the intention toward health-related internet use was mediated by behavioral attitude [20]. Thus, we posed the following hypothesis:

- H9: Health consciousness has a positive effect on the attitude of patients toward online inquiry in internet hospitals.

As well as health consciousness, PSD is a driving factor for health-seeking behaviors [18]. PSD could be defined as patients’ perception of the degree of seriousness of their untreated diseases, including opinions of clinical consequences and potential social consequences [27]. A higher perceived health risk of the disease consequences leads to a greater motivation to adopt a health-oriented behavior for access to health services. This hypothesis has been tested in several studies [19,20]. Several other studies on mHealth services also found that the PSD has a positive effect on behavioral intention [18,27]. In China, people usually tend to go to traditional high-level hospitals even for mild symptoms, owing to their lack of confidence in the quality of health care provided by primary care institutions [3]. When individuals perceive a more serious health threat, they might tend to consider the online inquiry service provided by internet hospitals challenging for the purpose of diagnosing or treating their diseases, and thus, they are likely to seek high-quality health services in traditional large hospitals rather than in internet hospitals. Thus, we posed the following hypothesis:

- H10: PSD has a negative effect on the patients’ PBC of online inquiry in internet hospitals.

**Figure 1:**
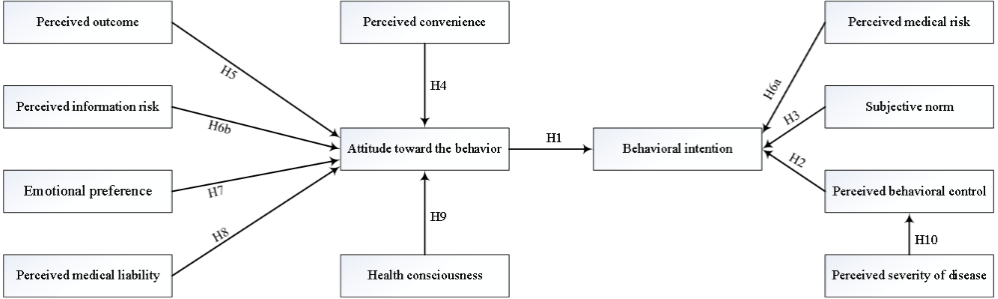
Structural research model.

The 11 research hypotheses, with reference to the results of eliciting salient beliefs from patient interviews, are summarized in the research model (Figure 1).

## Methods

### Development of the Study Questionnaire

We developed a survey questionnaire for the research model. We interviewed 19 patients effectively according to the connotation requirements of the TPB [41]; the results of eliciting salient beliefs from patient interviews were used to design the questionnaire items, and the items were also discussed and approved by a focus group. The questionnaire items (Textbox 1) were measured with a 5-point Likert scale ranging from “strongly disagree” (1) to “strongly agree” (5). We then performed a presurvey to validate the designed questionnaire.

**Measurement items of the constructs.**

**Perceived convenience**

1. I think it is convenient to use the online inquiry services provided by internet hospitals anywhere without going to the hospital in person.
2. I think that internet hospitals could provide a vast amount of medical information and help me find well-known hospitals or doctors across the country.
3. I think it is time-saving to make an online inquiry through internet hospitals.
4. I think that using the online inquiry services provided by internet hospitals could be helpful to save expenses such as transportation and lodging fees.
5. I think that using the online inquiry services provided by internet hospitals could be helpful to decrease losses such as the loss of normal income due to sick leaves.

**Perceived outcome**

1. I think that the online inquiry services provided by internet hospitals are helpful to accurately understand the conditions.
2. I think that effective treatment recommendations could be obtained through the online inquiry services provided by internet hospitals.
3. I think that effective medication guidance could be obtained through the online inquiry services provided by internet hospitals.

**Perceived medical risk**

1. I am worried that physicians in internet hospitals pay less attention to my conditions.
2. I have doubts about the professionalism of internet hospitals or their physicians.
3. I have doubts about the authenticity of internet hospitals or their physicians.
4. I am worried that I may not be able to clearly explain my conditions to physicians when using online inquiry services provided by internet hospitals.
5. I am worried that, because the online inquiry services provided by internet hospitals cannot provide medical examinations, it may result in misdiagnosis and missed diagnosis.

**Perceived information risk**

1. I am worried that I would be deceived during the use of the online inquiry services provided by internet hospitals.
2. I am worried that the personal information could be disclosed after using the online inquiry services provided by internet hospitals.

**Emotional preference**

1. I think that using the online inquiry services provided by internet hospitals could avoid the tension associated with face-to-face communication with physicians.
2. I think that using the online inquiry services provided by internet hospitals could be helpful to protect personal information and avoid the embarrassment associated with face-to-face communication with physicians.

**Perceived medical liability**

1. I think the relevant laws and regulations about the online inquiry services provided by internet hospitals are at an imperfect stage.
2. I think it is difficult to divide the liability of medical accidents encountered between patients, physicians, hospitals, and network platforms when using the online inquiry services provided by internet hospitals.
3. I think it is difficult to protect my legal rights when a medical accident occurs while using the online inquiry services provided by internet hospitals.

**Attitude toward the behavior**

1. Overall, I think using the online inquiry services provided by internet hospitals is a better experience than those provided by traditional hospitals.
2. Overall, I think using the online inquiry services provided by internet hospitals is helpful for disease treatment.
3. Overall, I think it is meaningful to using the online inquiry services provided by internet hospitals.

**Subjective norm**

1. Both family and friends around me think that I should use the online inquiry services provided by internet hospitals.
2. Patients around me think that I should use the online inquiry services provided by internet hospitals.
3. Web friends think that I should use the online inquiry services provided by internet hospitals.
4. The government has been advocating the use of online inquiry services provided by internet hospitals.

**Health consciousness**

1. I am aware of and very concerned about my health problems.
2. I will try to manage and improve my wellness.

**Perceived severity of disease**

1. I do not think my health problems are serious.

**Perceived behavioral control**

1. I have appropriate hardware equipment (such as smartphone and computer) to use the online inquiry services provided by internet hospitals.
2. I could independently complete the online operation process of using the online inquiry services provided by internet hospitals.
3. I could clearly distinguish the valid and invalid information about the online inquiry services provided by internet hospitals.

**Behavioral intention**

1. If I am sick, I will choose an internet hospital for online inquiry.
2. If someone around me requires health services, I will recommend the online inquiry services provided by internet hospitals to them.

In the presurvey analysis, we selected the outpatients who visited a tertiary public hospital, patients who bought medicines in a large drugstore, and patients from communication groups with chronic diseases (including diabetes, hypertension, and heart disease) as respondents. The presurvey sample size was often suggested to be 5-10 times the number of observed variables of the scale, or greater than or equal to 100 [42], and thus, we selected 150 patients by convenience sampling, and 128 patients were volunteered to participate in the presurvey during December 2019. Finally, a validation sample of 117 patients was used for testing reliability and validity of the designed questionnaire. Reliability was measured using Cronbach alpha. If the Cronbach alpha of a construct increases significantly after the removal of an item, indicating that the item reduces the internal consistency of its measurement, the item should be further revised or removed. The Cronbach alpha of all constructs should be higher than .70 [43,44]. We used exploratory factor analysis with varimax-rotated components to measure the validity of the designed questionnaire, and its cumulative total of variance and factor loadings were used to assess the construct validity. If the cumulative total of variance of the principal components selected accounted for more than 70% of the total variance, the composition of the principal components as exogenous constructs was consistent with the constructs of the designed questionnaire, and if each item had a factor loading value of 0.50 or higher on one of the principal components but factor loading values below 0.50 on others, the validity of the designed questionnaire was considered acceptable, and no items were removed.

No items were revised or removed on the basis of the analysis result of the presurvey, and thus, the designed questionnaire with acceptable reliability and validity could be further used for data collection. Structural equation modeling (SEM) was then used to verify the research model. Data on participants’ demographic characteristics such as gender, age, education level, marital status, living alone or with others, residence, monthly average income, types of medical insurance, and online inquiry experience were also collected.

### Data Collection

The target population included patients with chronic diseases including diabetes, hypertension, and heart disease, selected using the cluster sampling technique. We used the developed questionnaire to create an electronic questionnaire using the web-based survey tool wenjuanxing [45], a widely used website for conducting surveys in China. To collect enough sample data, the sample size was determined by general guidelines for SEM, and the samples were recommended to be at least 10-15 times as many as the items of the scale [46]. From February 12-29, 2020, we sent the survey link to Tencent QQ groups, WeChat groups, and Baidu Post Bar of patients with the aforementioned chronic diseases. Prior to the beginning of the survey, an informed consent of the managers of these patient social platforms was also requested and obtained. Moreover, we selected some general practitioners working in the community health center and physicians working in the tertiary hospital via convenience sampling, and these doctors were devoted to the treatment and management of the aforementioned chronic diseases. We then sent the survey website link to the doctors, and subsequently, they sent the website link to the targeted patient communication groups via WeChat. Participants could click on the website link using their phones to access and complete the electronic questionnaire. Before the survey, we introduced the purpose of the survey, provided the definition of internet hospitals, and guaranteed that the survey data would not be used for other purposes. After an individual’s consent was obtained, the survey was conducted accordingly. A WeChat, Tencent QQ, or Baidu Post Bar account and mobile Internet Protocol address could be used to complete the questionnaire only once. The study was approved by the Medical Ethics Committee of Tongji Medical College of Huazhong University of Science & Technology.

### Statistical Analysis

Descriptive statistics were used to analyze the demographic characteristics of the respondents. Patients’ intention to use the online inquiry services provided by internet hospitals was measured using two items (Textbox 1), with a higher score indicating elevated adoption intention. These respondents were divided into a younger group (younger than 50 years) and an older group. An independent *t* test was further used to evaluate the differences among adoption intention scores between the younger and the older groups.

SEM was used to verify the research model. Before evaluating the structural model, we assessed the measurement model to evaluate the reliability, convergent validity, and data fit indexes. We measured the reliability using the Cronbach alpha and composite reliability (CR). Values of .70 or higher for Cronbach alpha and values of 0.6 or higher for CR for all constructs were considered acceptable [8,43,44]. Convergent validity was measured using the average variance extracted (AVE) and standard loadings. The AVE of each construct and all the standard loadings should be greater than 0.50 [8,16,19]. The model fit was generally considered acceptable when the indexes met the criteria including χ^2^ / df<3, root mean square error of approximation<0.08, goodness-of-fit index (GFI)>0.90, adjusted GFI>0.90, Tucker-Lewis index>0.90, comparative fit index>0.90, and normed fit index>0.90 [13,27,47,48]. Otherwise, the research model was further revised. Moreover, we performed a bootstrap analysis with 1000 bootstrap bias-corrected samples to assess the direct, indirect, and total effect of variables (constructs) on the endogenous variables (ie, attitude and behavioral intention toward using online inquiry) [8,48]. Data analysis was done using SPSS V.24 and AMOS V.21 (IBM Corp).

## Results

### Sample Characteristics

In total, 812 responses (online survey) were received; of these, 174 responses were excluded because they contained identical answers to all questions, displayed certain logical contradictions, or because the time taken to answer the questionnaire was less than 100 seconds. The demographic characteristics of the 638 participants are shown in Table 1. Among these participants, 332 (52.0%) were aged 19-29 years, and 197 (30.9%) experienced the online inquiry services provided by internet hospitals. On average, the patients’ intention to use the online inquiry services provided by internet hospitals (min 1, max 5) was moderate, with a mean score of 3.21 (SD 0.84), and differences were not observed between younger patients and older patients (mean 3.19, SD 0.84 vs mean 3.36, SD 0.82; *P*=.10).

**Table 1:** Demographic characteristics of the participants (N=638).

Characteristics		Value, n (%)
**Gender**		
	Male	241 (37.8)
	Female	397 (62.2)
**Age (years)**		
	≤18	14 (2.2)
	19-29	332 (52.0)
	30-39	109 (17.1)
	40-49	110 (17.2)
	50-59	62 (9.7)
	≥60	11 (1.7)
**Education level**		
	Up to secondary school	241 (37.8)
	College	85 (13.3)
	Undergraduate	206 (32.3)
	Postgraduate and higher	106 (16.6)
**Marital status**		
	Married	325 (50.9)
	Unmarried	313 (49.1)
**Living alone or with others**		
	Living alone	70 (11.0)
	Living with family	510 (79.9)
	Living with friends	58 (9.1)
**Residence**		
	Rural	263 (41.2)
	Urban	375 (58.8)
**Monthly average income, ¥ (US $)**		
	<2000 (285.40)	252 (39.5)
	2000 (285.40)-5000 (713.60)	243 (38.1)
	5001 (713.80)-8000 (1141.80)	91 (14.3)
	>8000 (1141.80)	52 (8.2)
**Types of medical insurance**		
	Basic medical insurance for urban and rural residents	362 (56.7)
	Basic medical insurance for urban employees	196 (30.7)
	Commercial medical insurance	24 (3.8)
	Others	56 (8.8)
**Online inquiry experience**		
	Yes	197 (30.9)
	No	441 (69.1)

### Measurement Model Testing

Table 2 shows the results of the measurement model. The Cronbach alpha and CR of each construct were higher than the recommended values, indicating excellent reliability. The AVEs of constructs, except PMR, SN, and PBC, and all the standard loadings were greater than the recommended values, indicating acceptable convergent validity. Specifically, for example, the values of Cronbach alpha and CR in PC were .866 and .877, respectively, and higher than the recommended values of .70, indicating the measurement model had excellent reliability; moreover, the AVE was 0.597 and greater than the threshold value of 0.50, indicating the measurement model had acceptable convergent validity.

The goodness-of-fit results of the research model are shown in Table 3. All fit indexes were below the recommended values, indicating that the data collected did not fit with the research model, and thus further revision of the research model was needed.

**Table 2:** Statistical results of the research model

Constructs and items		Standard loadings	*P* value	Score, mean (SD)	Cronbach alpha	CR^a^	AVE^b^
**PC^c^**					.866	0.877	0.597
	PC1^d^	0.762	N/A^e^	3.82 (0.94)			
	PC2	0.711	<.001	3.97 (0.77)			
	PC3	0.782	<.001	3.98 (0.83)			
	PC4	0.757	<.001	4.11 (0.75)			
	PC5	0.753	<.001	3.91 (0.79)			
**PO^f^**					.850	0.773	0.533
	PO1	0.755	N/A	3.22 (0.96)			
	PO2	0.848	<.001	3.53 (0.86)			
	PO3	0.833	<.001	3.57 (0.82)			
**PMR^g^**					.847	0.794	0.438
	PMR1	0.663	N/A	2.59 (0.94)			
	PMR2	0.816	<.001	2.75 (0.91)			
	PMR3	0.832	<.001	2.76 (0.93)			
	PMR4	0.648	<.001	2.35 (0.94)			
	PMR5	0.659	<.001	2.12 (0.83)			
**PIR^h^**					.796	0.703	0.545
	PIR1	0.888	N/A	2.54 (0.96)			
	PIR2	0.747	<.001	2.30 (0.90)			
**EP^i^**					.770	0.712	0.562
	EP1	0.649	<.001	3.41 (0.90)			
	EP2	0.946	N/A	3.48 (0.89)			
**PML^j^**					.840	0.763	0.518
	PML1	0.718	<.001	2.09 (0.79)			
	PML2	0.845	N/A	2.15 (0.81)			
	PML3	0.832	<.001	2.13 (0.81)			
**AB^k^**					.813	0.887	0.723
	AB1	0.680	N/A	3.61 (0.72)			
	AB2	0.735	<.001	3.77 (0.65)			
	AB3	0.755	<.001	3.81 (0.66)			
**SN^l^**					.858	0.796	0.496
	SN1	0.763	N/A	3.28 (0.85)			
	SN2	0.880	<.001	3.11 (0.90)			
	SN3	0.802	<.001	3.11 (0.91)			
	SN4	0.658	<.001	3.25 (0.86)			
**HC^m^**					.801	0.790	0.661
	HC1	1.000	<.001	4.05 (0.69)			
	HC2	0.670	N/A	4,12 (0.63)			
**PSD^n^**					—^o^	0.630	0.630
	PSD1	0.744	N/A	2.54 (0.91)			
**PBC^p^**					.731	0.680	0.422
	PBC1	0.641	N/A	3.87 (0.82)			
	PBC2	0.863	<.001	3.74 (0.84)			
	PBC3	0.599	<.001	3.35 (0.86)			
**BI^q^**					.846	0.695	0.533
	BI1	0.796	N/A	3.19 (0.88)			
	BI2	0.831	<.001	3.23 (0.92)			

^a^CR: composite reliability.

^b^AVE: average variance extracted.

^c^PC: perceived convenience.

^d^Numbers refer to the numbered lists under each construct in Textbox 1.

^e^N/A: not applicable.

^f^PO: perceived outcome.

^g^PMR: perceived medical risk.

^h^PIR: perceived information risk.

^i^EP: emotional preference.

^j^PML: perceived medical liability.

^k^AB: attitude toward the behavior.

^l^SN: subjective norm.

^m^HC: health consciousness.

^n^PSD: perceived severity of disease.

^o^Not available because of only an item.

^p^PBC: perceived behavioral control.

^q^BI: behavioral intention.

**Table 3:** Goodness-of-fit results of the research model.

Fit indexes	χ^2^ / df	RMSEA^a^	GFI^b^	AGFI^c^	TLI^d^	CFI^e^	NFI^f^
Research model	5.185	0.0813	0.767	0.732	0.786	0.803	0.768
Recommended value	<3	<0.08	>0.90	>0.90	>0.90	>0.90	>0.90

^a^RMSEA: root mean square error of approximation.

^b^GFI: goodness-of-fit index.

^c^AGFI: adjusted goodness-of-fit index.

^d^TLI: Tucker-Lewis index.

^e^CFI: comparative fit index.

^f^NFI: normed fit index.

### Model Modification

To improve the goodness of fit of the research model, hypothesis paths of H3, H5, H8, and H10 were removed. Furthermore, the constructs including PML, PO, and SN were removed. Considering that the AVEs of the constructs PMR and PBC were considerably lower than the recommended values, indicating the measurement indexes with interference, the items including PMR2, PMR3, and PBC3 were removed. Moreover, the items including attitude toward behavior 1, PC4, and PC5 were removed on the basis of a focus group discussion. Additionally, the relationships between PBC and PC, between PBC and PMR, between PSD and PMR, between PSD and PBC, between PMR and PIR, between PMR and EP, and between EP and PC were established.

As shown in Table 4, after modification, the goodness of fit of the research model was improved. All fit indexes were greater than the recommended values, indicating that the eight-construct model was a good fit for the data collected.

**Table 4:** Goodness-of-fit results of the revised research model.

Fit indexes	χ^2^ / df	RMSEA^a^	GFI^b^	AGFI^c^	TLI^d^	CFI^e^	NFI^f^
Research model	2.837	0.054	0.942	0.920	0.933	0.946	0.920
Recommended value	<3	<0.08	>0.90	>0.90	>0.90	>0.90	>0.90

^a^RMSEA: root mean square error of approximation.

^b^GFI: goodness-of-fit index.

^c^AGFI: adjusted goodness-of-fit index.

^d^TLI: Tucker-Lewis index.

^e^CFI: comparative fit index.

^f^NFI: normed fit index.

### Structural Model Testing

**Figure 2:**
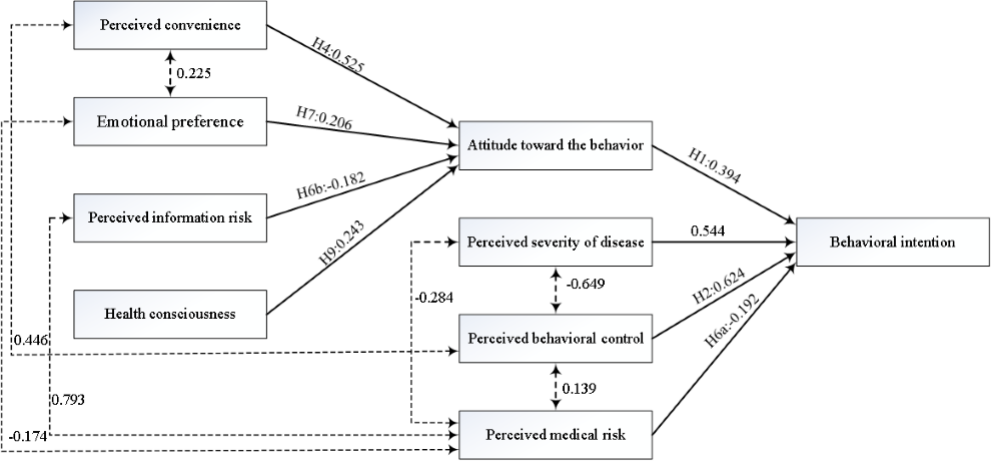
Results of the final research model.

Table 5 shows that three (H3, H5, and H10) of the eleven research hypotheses were rejected. The standardized path coefficients of all other relationships were significant at *P*<.05. The relationships among constructs in the final research model are illustrated in Figure 2.

**Table 5:** Hypothesis testing results of the research model

Hypothesis paths	Standardized path coefficients	*P* value	Results
H1^a^ Attitude toward the behavior (+^b^) → Behavioral intention	0.394	<.001	H1 supported
H2 Perceived behavioral control (+) → Behavioral intention	0.624	<.001	H2 supported
H3 Subjective norm (+) → Behavioral intention	—^c^	—	H3 not supported
H4 Perceived convenience (+) → Attitude toward the behavior	0.525	<.001	H4 supported
H5 Perceived outcome (+) → Attitude toward the behavior	—	—	H5 not supported
H6a Perceived medical risk (–^d^) → Behavioral intention	–0.192	<.001	H6a supported
H6b Perceived information risk (–) → Attitude toward the behavior	–0.182	<.001	H6b supported
H7 Emotional preference (+) → Attitude toward the behavior	0.206	<.001	H7 supported
H8 Perceived medical liability (–) → Attitude toward the behavior	—	—	H8 not supported
H9 Health consciousness (+) → Attitude toward the behavior	0.243	<.001	H9 supported
H10 Perceived severity of disease (–) → Perceived behavioral control	—	—	H10 not supported

^a^H: hypothesis.

^b^+: positive effect.

^c^Hypothesis paths of H3, H5, H8, and H10 were removed in the revised model.

^d^–: negative effect.

Table 6 shows the total, direct, and indirect effects (standardized path coefficients) of the model variables (constructs) on attitude toward the behavior and behavioral intention, respectively. PC, PIR, EP, and health consciousness explained 45.9% of the variance in attitude toward the behavior. Among these four variables, PC had the strongest effect on attitude toward the behavior (β=.525, *P*=.002). EP and health consciousness had moderate effects on attitude toward the behavior (β=.206, *P*=.002 and β=.243, *P*=.002, respectively). Additionally, PIR had a slightly negative direct effect on attitude toward the behavior (β=–.182, *P*=.001).

Further, PC, PIR, EP, and health consciousness had indirect effects on behavioral intention, and these effects were mediated by attitude toward the behavior. PC had the highest indirect effect on behavioral intention (β=.207, *P*=.001) among the four constructs.

Overall, attitude toward the behavior, PMR, PSD, and PBC explained 60.5% of the variance in behavioral intention. PBC and PSD had the highest total effects on behavioral intention (β=.624, *P*=.004 and β=.544, *P*=.003, respectively). Attitude toward the behavior had a moderate total effect on behavioral intention (β=.394, *P*=.002), whereas PMR had a slightly negative total effect on behavioral intention (β=–.192, *P*=.005).

Within expectations, PMR had the strongest positive relationship with PIR (*r*=0.793, *P*=.001). Moreover, PMR had a slightly negative relationship with EP and PSD (*r*=–0.174, *P*=.002 and *r*=–0.284, *P*=.002, respectively) but a positive relationship with PBC (*r*=0.139, *P*=.002). Additionally, PBC had a moderate positive relationship with PC (*r*=0.446, *P*=.001) but was strongly and negatively correlated with PSD (*r*=–0.649, *P*=.002). PC also had a slightly positive relationship with EP (*r*=0.225, *P*=.002).

**Table 6:** Total, direct, and indirect effects of the model variables.

Variables		AB^a^ (*R*^2^=45.9%)		BI^b^ (*R*^2^=60.5%)	
		β	*P* value	β	*P* value
**PC^c^**					
	Direct	.525	.002	—^d^	—
	Indirect	—	—	.207	.001
	Total	.525	.002	.207	.001
**PMR^e^**					
	Direct	—	—	–.192	.005
	Indirect	—	—	—	—
	Total	—	—	–.192	.005
**PIR^f^**					
	Direct	–.182	.001	—	—
	Indirect	—	—	–.072	.001
	Total	–.182	.001	–.072	.001
**EP^g^**					
	Direct	.206	.002	—	—
	Indirect	—	—	.081	.001
	Total	.206	.002	.081	.001
**HC^h^**					
	Direct	.243	.002	—	—
	Indirect	—	—	.096	.001
	Total	.243	.002	.096	.001
**PSD^i^**					
	Direct	—	—	.544	.003
	Indirect	—	—	—	—
	Total			.544	.003
**PBC^j^**					
	Direct	—	—	.624	.004
	Indirect	—	—	—	—
	Total			.624	.004
**AB**					
	Direct	—	—	.394	.002
	Indirect	—	—	—	—
	Total	—	—	.394	.002

^a^AB: attitude toward behavior.

^b^BI: behavioral intention.

^c^PC: perceived convenience.

^d^Not available.

^e^PMR: perceived medical risk.

^f^PIR: perceived information risk.

^g^EP: emotional preference.

^h^HC: health consciousness.

^i^PSD: perceived severity of disease.

^j^PBC: perceived behavioral control.

## Discussion

### Principal Findings

Our study shows that the patients’ intention to use the online inquiry services provided by internet hospitals was relatively low, with room for development. Internet hospitals in China show an emerging trend for adequately fulfilling people’s demands for health services [9,10]. Thus, the determinants of patients’ intention to use online inquiry services provided by internet hospitals are needed that help provide opportunities to promote the development of internet hospitals. Our findings show the patients’ intention to use the online inquiry services provided by internet hospitals was determined by the following factors: attitude toward the behavior, PBC, PSD, PC, PIR, PMR, health consciousness, and EP.

Our study found that two TPB factors (ie, attitude toward the behavior and PBC) had significant effects on patients’ intention to use the online inquiry services provided by internet hospitals, whereas the factor (ie, SN) had an insignificant effect on behavioral intention. PBC was the most important determinant of patients’ intention to use the online inquiry services provided by internet hospitals. Several studies on mHealth services also indicated PBC was the major determinant of behavioral intention [24,26,27]. If patients find it easy to use online inquiry services, their willingness toward online inquiry services provided by internet hospitals will be stronger. Many studies found that the perceived ease of use of mHealth technology is a determinant of patients’ adoption intention toward mHealth services [8,21,49,50], especially among older adults [16]. Similar to mHealth service, the ease of using online inquiry services provided by internet hospitals must be promoted, thereby potentially improving the PBC of users in using these services. Therefore, internet hospitals should be patient-centered, further optimize online service delivery process to improve the ease of using online inquiry services, and provide patients with multiple convenient and easy-to-use channels for online inquiry, such as mHealth service apps and WeChat official accounts.

PSD had a strong positive total effect on patients’ intention to use the online inquiry services provided by internet hospitals, and this effect was also direct but not mediated by PBC. This finding is consistent with the findings of a study by Zhang et al [27] regarding mHealth service adoption in China. Another study by Liu et al [51] also found a positive effect of PSD on propensity of users in online health care communities to seek health information. This finding suggests that perceived disease severity motivates patients to adopt online inquiry services provided by internet hospitals. Internet hospitals, owing to the integration of a large number of high-quality medical resources across the country could competently fulfill the increasing patient demands for high-quality health services. It is difficult for patients in rural and remote areas of China to access high-quality health services [4,5]. Thus, internet hospitals should be dedicated to enhance the awareness of individuals regarding disease vulnerability and emphasize the advantages of online inquiry services to change the indifferent attitude and distrust of certain people toward the online inquiry services provided by internet hospitals. With the increasing health service requirements of patients, physicians tend to have a heavier workload [44], and unreasonable expectations regarding online services may further increase the physicians’ workload. Therefore, internet hospitals should also ensure a reasonable assembly of high-quality experts and coordinate their online and offline medical resources. Ensuring a reasonable assembly of high-quality medical resources in internet hospitals will not only optimize the physicians’ workload but also meet the increasing patient demands for renowned experts, thereby promoting patients’ willingness toward online inquiry services provided by internet hospitals.

PC also had a moderate positive total effect on patients’ intention to use online inquiry services provided by internet hospitals. This finding is in line with the findings of a study of Valikodath et al [29] on patients’ willingness toward telemedicine for diabetic retinopathy. Another study by Clevenger et al [52] also demonstrated that PC was positively associated with acceptance of adolescent vaccination outside of the traditional medical home. This finding suggests the need for internet hospital managers to identify the gaps in convenience of health care service delivery and further improve the organizational service delivery design. During the special survey time called “Wuhan on lockdown,” this policy might contribute to a greater intention of patients in the Wuhan area to use online inquiry services provided by internet hospitals owing to the inconvenience of seeking health services in traditional hospitals. With the normalization of the prevention and control of the COVID-19 epidemic, service convenience has become an important topic in the context of health care, and internet hospitals play an increasingly important role in delivering health services to the public. Therefore, internet hospitals should also promote and achieve one-step health care services via the integration of medical service, medicine, and medical insurance. Improving service convenience will help people reduce the risks of nosocomial cross-infection and, thus, benefit the promotion of patients’ intention to use the online inquiry services provided by internet hospitals.

Although the indirect effect of PIR on patients’ intention to use online inquiry services provided by internet hospitals was weak (and this effect was mediated by attitude toward the behavior), PMR had a significantly negative stronger direct effect on behavioral intention. Similarly, several studies on mHealth services found the negative effect of perceived privacy risk on behavioral intention [13,53]. Another study by Zhang et al [8] also demonstrated the negative effect of perceived privacy risk on patients’ intention to use diabetes management apps. With the rapid development of health information technology, patients are increasingly aware of and concerned about their personal information protection such as privacy protection on health care–seeking data. Although our research found that PIR had only a weak effect on patients’ intention to use the online inquiry services provided by internet hospitals, solid patient measures to protect patient information are needed. Additionally, our research found that PMR had a stronger effect on patients’ intention to use online inquiry services provided by internet hospitals. However, to date, the Chinese government has not established an effective quality control system for internet hospitals, and related regulations from traditional hospitals are often used for the management of medical behaviors in internet hospitals, although the government have realized the problem [12,15], which could lead to an increase in probability of medical risks, threatening patient safety [38], thereby potentially affecting risk-conscious patients’ intention to use the online inquiry services. Thus, to ensure patient safety regarding the use of online inquiry services provided by internet hospitals, the relevant quality control standards and regulations for internet hospitals should be further developed and improved.

Health consciousness had slightly positive indirect effects on patients’ intention to use the online inquiry services provided by internet hospitals, and these effects were mediated by attitude toward the behavior. A previous study by Ahadzadeh et al [20] suggested that health consciousness positively affected patients’ intention of the internet use for health-related purposes. Several studies concerning disease information-seeking behaviors also demonstrated an effect of health consciousness on behavioral intention [54,55]. Patients with a higher level of health consciousness are more likely to be aware of and concerned about their wellness [56], and if they believe that their health service requirements can be adequately fulfilled by the online inquiry services provided by internet hospitals, their willingness toward online inquiry will be stronger. With the increasing health awareness of people in China, their willingness toward online inquiry services provided by internet hospitals will be further improved.

Our study also found that EP had a slightly positive total effect on patients’ intention to use the online inquiry services provided by internet hospitals. Emotion is an essential element of human behavior. Regarding health behaviors, some patients with genital diseases or patients who are sensitive about a certain disease have more awareness about self-precautionary measures and privacy protection or are more unwilling to disclose their condition to others, eventually affecting their help-seeking intentions. Such unfavorable results have been found in several studies on patients with mental health problems [35,36]. With internet hospitals, such patients could be provided with a more confidential approach of seeking health services through graph-text, voice, and video services [9], which protects their privacy and fulfills their emotional requirements to a certain extent; thus, patients with EPs would be more willing to gain access to the online services provided by internet hospitals.

### Limitations

This study has several limitations. First, among the twelve constructs, PSD included only one item, which may have resulted in a measurement error. Second, although the research model was developed based on the TPB, some factors influencing patients’ intention to use online inquiry services provided by internet hospitals may not have been identified; for example, “medical expenses” was included in PC but deleted in the revised model; the medical service price is an important determinant of patients’ intention to accept a medical service in traditional hospitals, especially whether this service is covered by medical insurance payment [57], but was not as a factor included into the research model, and thus, continuous research is needed to help further explore these determinants. Third, online survey was used as the data collection method owing to the COVID-19 epidemic, and the target population included individuals with chronic diseases such as diabetes, hypertension, and heart disease; although the age of patients with chronic diseases was usually old, the younger patients in targeted patient communication groups might be more willing to participate in the online survey; moreover, we used cluster sampling rather than proportional sampling, and although differences were not observed between younger patients and older patients, our results may have been biased by the age distributions. Further, data collection was self-reported by patients via online survey, which might have a recall bias. In addition, to improve the goodness of fit of the research model, a few constructs including PML, PO, and SN were removed, which may have produced an insignificant result; therefore, further research is necessary to identify whether these constructs significantly affect patients’ intention to use online inquiry services provided by internet hospitals.

### Conclusions

PBC and PSD are the most important determinants of patients’ intention to use online inquiry services provided by internet hospitals. Therefore, internet hospitals should further optimize the design of online service delivery and ensure a reasonable assembly of high-quality experts, which will benefit the promotion of patients’ adoption intention toward online inquiry services for health purposes. PC, EP, and perceived risks also have effects on behavioral intention. Therefore, the relevant quality control standards and regulations for internet hospitals should be further developed and improved, and the measures to protect personal information should be strengthened to ensure patient safety. Our study supports the use of the planned behavior theory in explaining patients’ intention to use the online inquiry services provided by internet hospitals.

## Abbreviations

AVE: average variance extracted
CR: composite reliability
EP: emotional preference
GFI: goodness-of-fit index
H: hypothesis
mHealth: mobile health
PBC: perceived behavioral control
PC: perceived convenience
PIR: perceived information risk
PML: perceived medical liability
PMR: perceived medical risk
PO: perceived outcome
PSD: perceived severity of disease
SEM: structural equation modeling
SN: subjective norm
TPB: theory of planned behavior
